# The association between estrogen receptor 2 gene polymorphism and complexity of coronary artery disease: an analysis in elective percutaneous coronary intervention patients

**DOI:** 10.1186/s12872-021-02088-1

**Published:** 2021-06-04

**Authors:** Farzaneh Foroughinia, Pooyan Dehghani, Mehdi Dianatpour, Arghavan Amiri, Iman Jamhiri, Parisa Ghasemiyeh

**Affiliations:** 1 Department of Clinical Pharmacy, School of Pharmacy, Shiraz University of Medical Sciences, Shiraz, Iran; 2 Cardiovascular Research Center, Shiraz University of Medical Sciences, Shiraz, Iran; 3 Stem Cell Technology Research Center, Shiraz University of Medical Sciences, Shiraz, Iran; 4 Department of Medical Genetics, School of Medicine, Shiraz University of Medical Sciences, Shiraz, Iran; 5 Student Research Committee, Shiraz University of Medical Sciences, Shiraz, Iran

**Keywords:** Cardiovascular disease, Estrogen receptor Beta, Polymorphism, SYNTAX score

## Abstract

**Background:**

One of the most common causes of death in the world is coronary artery disease (CAD). Estrogen, the most important early sex hormones in women, plays an important role in the risk reduction of cardiovascular disease (CVD). Expression of estrogen as well as its receptors including estrogen receptor alpha (ER1) and estrogen receptor beta (ER2) might have an association with the severity or the complexity of CAD. Since most articles have focused on the relationship between ER1 gene polymorphism and CAD, in this study, we aimed to evaluate the association of two ER2 gene polymorphisms, rs4986938 (AluI) and rs1256049 (RsaI), with the severity of CAD.

**Methods:**

148 patients with confirmed CAD who underwent elective percutaneous coronary intervention (PCI) were included in this study. Blood samples were collected before coronary angiography and ER2 gene polymorphisms were analyzed by the PCR–RFLP method. The STNTAX Score (SS), grading system for CAD complexity, was evaluated by an interventional cardiologist who was blinded to other data.

**Results:**

110 men and 38 women were participated in this study. Our results revealed a statistically significant relationship between SS and rs4986938 polymorphism of ER2 in men. In contrast, there was no association between rs1256049 genotypes and SS after performing regression analysis.

**Conclusions:**

Besides to the estrogen level, the genetic variation of its receptors might play an important role in the severity or the complexity of CAD. According to our results, rs4986938 polymorphism of ER2 gene may assert a pivotal role in the severity of CAD in men; however, this assumption needs to be proved in studies with a larger population.

**Supplementary Information:**

The online version contains supplementary material available at 10.1186/s12872-021-02088-1.

## Background

Cardiovascular disease (CVD) is considered as one of the major causes of death worldwide. Coronary artery disease (CAD) and cerebrovascular diseases are among the most common causes of deaths due to CVD [1]. According to the epidemiologic studies, premenopausal women have less risk for CVD in comparison with men with the same age that may be because of the protective effects of estrogen in the prevention of CVD [2]. Estrogen can serve its effect by binding to estrogen receptor α (ER1), estrogen receptor β (ER2), and G-protein-coupled estrogen receptor 30 (GPR30). The possible protective mechanism of estrogen against CVD would be related to its effect on angiogenesis, fibrosis, vascular function, and oxidative stress processes [3].

Estrogen could induce its cardioprotective effects through a receptor-based biological mechanism. Recent researches revealed that genetic variants have an important role in cardio- and cerbro-vascular diseases. For instance, ER1 is an important indicator of atheroprotective effect of estrogen on cardiovascular system. [4]. A study evaluated the association between ER1 single nucleotide polymorphism (SNP) and Intima-media thickness (IMT) in carotid artery, an important predictor of CVD, demonstrated a significant association between rs2228480 and rs3798758 with the IMT of carotid artery in Taiwanese women [5]. A case-cohort design on evaluation of the relationship between ER1 SNP and the risk of coronary heart disease (CHD) and stroke in Finnish population, revealed the significant association of rs2334693 polymorphism with the higher risk of CHD in men but not with ischemic stroke [6]. In addition, a significant association between ER1 gene polymorphism and low density lipoprotein (LDL) metabolism, an important predictor of atherosclerosis and CVD, was found in women [7]. Arterial stiffness enhancement and increased wave reflection are the other risk factors for CVD. It has been hypothesized that there is an association between arterial stiffness and genetic variance of ER1, ER2, and CYP19A1 (aromatase). Results of the American population study showed a strong relation between ER1 and ER2 gene polymorphisms and enhanced wave reflection but it is failed to show any association respected to CYP19A1 [8]. An additional table shows these studies in more detail [see Additional file 1].

The SYNTAX (Synergy between PCI with Taxus and Cardiac Surgery) score (SS) is an angiographic tool using to predict the coronary vasculature characteristics based on the number of lesions and also their complexity, location, and function. SS would be calculated through a computer program using 12 main questions which are about the dominance, number of lesions, segments involved per lesion, total occlusion, trifurcation, bifurcation, aorto-ostial lesion, severe tortuosity, length > 20 mm, heavy calcification, thrombus, and diffuse disease/small vessels. Higher SS would be a predictor of higher level of disease complexity, more challenging pharmacotherapy, and worse prognosis following coronary intervention [9].

Since most of studies have focused on ER1 and few papers have evaluated the role of ER2 in atherosclerotic diseases, in this study, we aimed to evaluate the association between two major SNPs of ER2 gene containing rs4986938 (AluI) and rs1256049 (RsaI) and the severity and the complexity of CAD expressed as SS.

## Methods

The study protocol was assessed and approved by the Ethics Committee of Shiraz University of Medical Sciences (SUMS, Iran) (No: IR.SUMS.REC.1397.369). Written consent form and permission for DNA analyses was obtained from all participants.

### Subjects

Of 200 patients, admitted to two tertiary care hospitals of SUMS (Ghalb-Al-Zahra and Nemazee hospitals) with the diagnosis of CAD from August 2018 to February 2019, 148 participants were recruited to the study (fifty-two patients were excluded due to lack of data and refusal to sign the consent form).

Inclusion criteria were age of 32–80 years old and confirmed diagnosis of CAD (> 50% luminal stenosis in at least one major coronary artery in angiography). Exclusion criteria include consumption of oral contraceptives and hormone replacement therapy during one month prior to the study, familial hypercholesterolemia, malignancy, schizophrenia, connective tissue disease, or chronic inflammatory diseases.

Patients who fulfilled the criteria were included into this project. Demographic and clinical data were documented for each patient according to the patients’ hospital record.

### Coronary angiography

Patients who were candidates for invasive strategy with coronary angiography were enrolled into the study. Patients received Aspirin, clopidogrel, and anticoagulation according to current guidelines prior to coronary angiography (aspirin, 325 mg loading dose and then 80 mg/d for the rest of their life and clopidogrel, 600 mg loading dose and then 75 mg/d for at least one year after PCI as well as weight-adjusted intravenous heparin with a target activated clotting time of 250–350 Sec before PCI [10]. Procedure was performed with local anesthesia and via radial or femoral approaches [11]. Ultravist® or Visipaque® was used as contrast agents.

Subjects with > 50% stenosis in at least one major epicardial artery greater than 1.5 mm were included into the study. Coronary angiographic data, including vessels' involvement, percentage of stenosis and SS were evaluated by an interventional cardiologist who was blinded to other data. SS was calculated using the official calculator from the SYNTAX score website [12].

The final plan of the management for patients was varied from medical therapy (11%) to percutaneous coronary intervention (PCI) (67%) and coronary artery bypass grafting in the remaining (22%).

Severe CAD is defined as ≥ 70% luminal stenosis in one major epicardial vessel or ≥ 50% stenosis in the left main coronary artery (LMD) and Multivessel disease (MVD) is defined as more than one coronary artery involvement with ≥ 70% stenosis [13, 14]. Patients were categorized into two groups according to the SYNTAX score: low risk (SS ˂ 23) and intermediate/ high risk (SS ≥ 23) [15].

### Biological samples and genotyping

In order to assess ER2 gene polymorphisms, blood samples were collected in the EDTA vacutainer tubes from all patients. Whole blood was stored at − 20 °C freezer until use. Genomic DNA for PCR was extracted from whole blood by DNA extraction kit (Yekta tajhiz, Iran) according to the manufacturer protocol.

Selected regions of ER2 gene were amplified by polymerase chain reaction (PCR) using specific primers listed in Table 1. All primers, used for the amplification of rs1256049 and rs4986938 polymorphisms regions in ER2 gene, were designed by the Primer 3 software and were obtained from metabion (metabion international AG, Martinsread, Germany). The reaction program for rs1256049 and rs4986938 polymorphisms amplification was as follows: initial denaturation for 4 min at 94°c, followed by 35 cycles of 94°c for 50 s, 66°c for 50 s, 72°c for 50 s and final elongation at 72°c for 7 min. The PCR reactions were performed in Veriti Thermal Cycler (Applied Biosystems, Foster City, CA, USA).

**Table 1:** Primers used in this study

Gene	Sequence	Band size (fragments obtained after digestion)
rs1256049 Forward	TTCTGAGCCGAGGTCGTAGT	582 bp (A: 293 bp + 289 bp; G: 582 bp)
rs1256049 Reverse	TGAATCCTTGGACCCAACTC	
rs4986938 Forward	GTGTGTGGTGGGACACAGAG	646 bp (A: 445 bp + 201 bp; G: 646 bp)
rs4986938 Reverse	AGGCCATTGAGTGTGGAAAC	

For RFLP, the PCR products of rs1256049 and rs4986938 polymorphisms were digested with RsaI (#ER1121, 5U at 37 °C for 16 h) and AluI (#ER0011, 5U at 37 °C for 16 h) (Fermentas), respectively. DNA fragments from RFLP were electrophoresed on 2% agarose gel to determine the rs1256049 and rs4986938 polymorphic patterns.

### Statistical analysis

Collected information of this study was analyzed using Statistical Package for Social Sciences (SPSS) for Windows software (version 16, Chicago, USA). All continuous variables were reported as the mean [standard deviation (SD)] and the differences between men and women were analyzed by independent samples t test. Categorical variables were showed as absolute number and percentages and were tested using chi-square test.

For comparison between ER2 genotypes and allele frequencies and SS or sex, chi-square test and Fisher’s exact test were applied. Multiple linear regression analysis was performed to assess the association between SS and conventional cardiovascular risk factors as well as investigated ER2 genotypes by genders.

## Results

Totally, 148 patients were enrolled into the study. The mean age of all patients was 59.06 (11.42). 110 (74.7%) men with the mean age of 57.62 (11.75) and 38 (25.3%) women with the mean age of 63.29 (9.28) were participated in our study.

Clinical baseline characteristics of patients were described in Table 2, dividing participants in two groups according to the sex. Both groups were similar in all variables with the exception of age (*p* = 0.01), glomerular filtration rate (GFR) (*p* =  < 0.001), frequency of hypertension (*p* =0.02), diabetes mellitus (DM) (*p* = 0.01), and active smoking (*p* = ˂0.001). In addition, no significant difference was found between sex and endpoints such as SS, severe CAD, and the number of diseased vessels. The distribution of SS in studied patients was reported in the supplementary file [see Additional file 2].

**Table 2:** Patients’ demographic and clinical characteristics

Variables	Total (n = 148)	Men (n = 110)	Women (n = 38)	*p* value
Age, years, mean (SD)	59.06 (11.42)	57.62 (11.75)	63.29 (9.28)	0.01
DM, N (%)	60 (40)	3 (33.6)	22 (57.9)	0.01
Hyperlipidemia, N (%)	68 (45.3)	46 (41.8)	21 (55.3)	0.15
Hypertension, N (%)	74 (49.3)	48 (43.6)	25 (35.8)	0.02
Current smoker, N (%)	68 (45.3)	64 (57.1)	4 (10.5)	< 0.001
Previous MI, N (%)	6 (4)	5 (4.5)	1 (2.6)	NA
BB-treated, N (%)	109 (72.7)	80 (71.4)	29 (76.3)	0.66
Statin-treated, N (%)	136 (91.1)	102 (92.7)	34 (89.5)	0.50*
ACE inhibitor-treated, N (%)	93 (62)	68 (60.7)	25 (65.8)	0.66
GFR (MDRD), ml/min/1.73m^2^, mean (SD)	77.16 (20.28)	81.41 (18.79)	64.61 (19.53)	< 0.001
Creatinine, mg/dL, mean (SD)	1.04 (0.22)	1.06 (0.21)	1.00 (0.25)	0.12
BUN, mg/dL, mean (SD)	16.31 (6.38)	15.86 (5.22)	17.64 (8.9)	0.15
SYNTAX Score, mean (SD)	18.61 (11.85)	17.86 (10.85)	19.79 (13.76)	0.38
SYNTAX Score, N (%)				
≥ 23	41 (27.3)	28 (25)	13 (34.2)	0.20
˂23	109 (72.7)	84 (75)	25 (65.8)	
Severe CAD, N (%)	122 (82.4)	90 (81.1)	32 (84.2)	0.74
Diseased vessel, N (%)				
One vessel	51 (34.5)	36 (33)	15 (39.5)	
Two vessel	56 (37.8)	44 (40.4)	12 (31.6)	0.66
Three vessel	40 (27)	29 (26.6)	11 (28.9)	
MVD, N (%)	74 (50)	56 (50.9)	18 (47.4)	0.71

*DM* diabetes mellitus, *MI* myocardial infarction, *BB* beta blocker, *ACEI* Angiotensin converting enzyme inhibitor, *GFR* glomerular filtration rate, *BUN* blood urea nitrogen, *CAD* coronary artery disease, *MVD* multi-vessel disease

*p values are from χ^2^ test

**Fig. 1:**
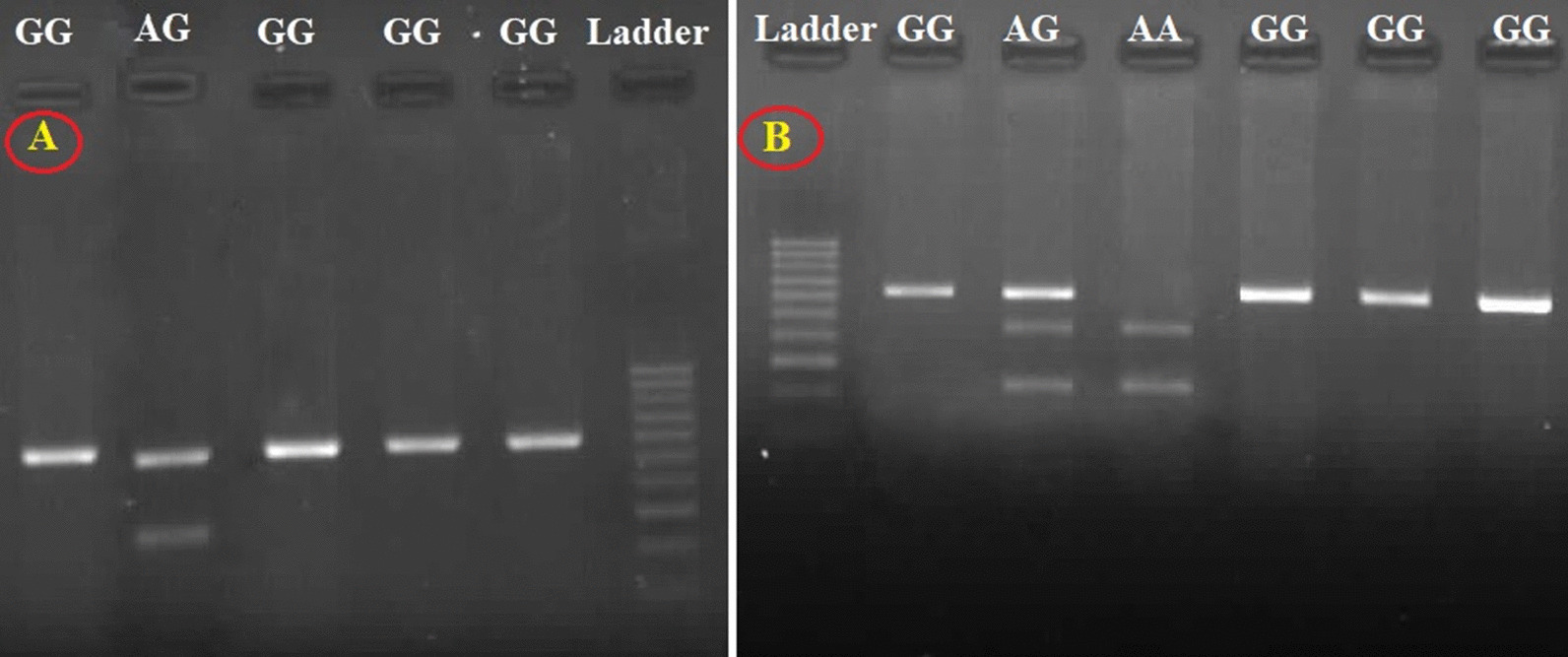
The gel electrophoresis results after enzymatic digestion. **A** Genoypes of rs1256049 polymorphisms. **B** Genoypes of rs4986938 polymorphisms. 100 bp ladder used. Full-length blots/gels are presented in Supplementary Figures [see Additional files 3 and 4]

All samples were examined for ER2 gene polymorphisms (rs1256049 and rs4986938). The gel electrophoresis results after enzymatic digestion are shown in Fig. 1. Of 148 samples analyzed for rs1256049 polymorphism, a statistically significant relationship was found between SS and rs1256049 genotypes of ER2 gene (*p* = 0.01) but not with rs1256049 alleles (*p* = 0.05). Of note, AG genotype was found to be the more prevalent ones in patients with SS ≥ 23. In contrast, there was no association between rs4986938 genotypes and alleles and SS. The same results were reported for the association between sex and rs1256049 and rs4986938 polymorphisms of ER2 gene (Table 3).

**Table 3:** Associations of sex and SYNTAX score with ER2 gene polymorphisms in patients with CAD

Receptor gene	SYNTAX score		*p* value	Gender		*p* value
	˂23 (n = 109)	≥ 23 (n = 41)		Men (n = 110)	Women (n = 38)	
*rs4986938, N (%)*						
GG	58 (53.2)	25 (61)	0.16	61 (54.5)	22 (57.9)	0.9
AG	37 (33.9)	15 (36.6)		40 (35.7)	12 (31.6)	
AA	14 (12.8)	1 (2.4)		11 (9.8)	4 (10.5)	
*Alleles, N (%)*						
A	63 (28.9)	19 (23.2)	0.32	64 (28.6)	18 (23.7)	0.41
G	155 (71.1)	63 (76.8)		160 (71.4)	58 (76.3)	
*rs1256049, N (%)*						
GG	101 (92.7)	32 (78)	0.01	98 (87.5)	35 (92.1)	0.82*
AG	7 (6.4)	9 (22)		13 (11.6)	3 (7.9)	
AA	1 (0.9)	0 (0)		1 (0.9)	0 (0)	
*Alleles, N (%)*						
A	9 (4.1)	9 (11)	0.05	15 (6.7)	3 (3.9)	0.53
G	209 (95.9)	73 (89)		209 (93.3)	73 (96.1)	

*p values are from χ^2^ test

According to the regression analysis, none of rs1256049 genotypes were associated with SS in both genders. Regarding rs4986938 polymorphism, there was a relationship between GG genotype and SS as well as AG genotype and SS in men; in contrast, no relationship was found in women. Table 4 presents cardiovascular risk factors and ER2 gene polymorphisms associated with SS by gender.

**Table 4:** Results of multiple linear regression analysis associated with SS values with adjustment for generally known cardiovascular risk factors and ER2 gene polymorphisms (rs1256049 and rs4986938)

Independent variables	Women (n = 38)		Men (n = 110)	
	Standardized β	95% C.I	Standardized β	95% C.I
Age	0.17	(− 0.25, 0.77)	0.07	(− 0.14, 0.26)
GFR (MDRD)	0.33	(− 0.01, 0.33)	0.03	(− 0.06, 0.09)
BUN	0.45	(0.15, 1.24)	0.14	(− 0.06, 0.64)
DM	− 0.19	(− 12.29, 2.12)	− 0.02	(− 4.30, 3.47)
Hypertension	− 0.09	(− 12.82, 7.71)	− 0.11	(− 5.98, 1.34)
Hyperlipidemia	0.16	(− 3.09, 11.97)	0.14	(− 0.74, 6.93)
Smoking	0.42	(− 10.97, 14.73)	0.09	(− 1.45, 5.43)
Number of diseased vessels	0.30	(− 2.87, 12.72)	0.36	(2.39, 7.82)
MVD	− 0.38	(− 23.63, 2.9)	− 0.19	(− 8.39, 0.26)
Sever CAD	0.14	(− 17.55, 7.53)	− 0.14	(− 8.69, 0.91)
rs1256049				
AA (reference)				
AG	NA	NA	0.22	(− 10.89, 25.83)
GG	− 0.14	(− 22.25, 7.97)	− 0.02	(− 18.51, 17.40)
rs4986938				
AA (reference)				
AG	− 0.09	(− 17.00, 12.00)	0.27	(0.08, 12.21)
GG	− 0.17	(− 19.17, 9.73)	0.28	(0.02, 12.15)

*GFR* glomerular filtration rate, *BUN* blood urea nitrogen, *DM* diabetes mellitus, *CAD* coronary artery disease, *MVD* multi-vessel disease, *NA* not assigned

## Discussion

In this study the association between ER2 gene polymorphisms, rs1256049 and rs4986938, and SS was evaluated in 148 Iranian patients with CAD. About 75% of our cases was men. The same gender distribution was found in our previous investigation in patients undergoing elective PCI [10]. According to our results, CVD risk factors such as hypertension and active smoking were more prevalent in men compared to women. Higher frequency for history of smoking in men is completely predictable since smoking is not a common habit among Iranian women due to cultural issues. Unfortunately, it is in a growing manner in recent years. In contrast, older age and more cases with diseases such as diabetic mellitus and chronic kidney disease (CKD) were reported to be more prevalent in studied women than men. Older ages in studied women is congruent with the fact that women experienced CVD at higher ages than men. In addition, more rates of CKD involvement in women may be assumed due to higher rate of DM among women than men in this study.

Results of polymorphism analysis revealed that there was no significant association between rs4986938 gene polymorphism and their alleles with SS, on the other hand; a significant association was found between rs1256049 genotypes and SS. However, after performing the multivariate adjustment of confounding factors such as age, sex, incidence of hypertension, diabetes, smoking, etc., it was indicated that the value of SS may be decreased in studied men with the presence of GG and AG at rs4986938. This revealed the higher risk of CAD complexity as well as the worser PCI outcome in patients with rs4986938 GG and AG genotypes in comparison to AA genotype. In contrast, this association was not reported in women. This result may be related to the small sample size of women in our study.

Even though several researches evaluated the association between ER gene polymorphisms and CVD prognosis, most of them worked on ER1 gene polymorphisms [16–19] and only few evidences investigated the association between ER2 gene polymorphisms and CVD progress [20].

According to the available databases, our study is the first one evaluated the association between SS and SNPs of ER2 gene. A recently published case–control study performed on Chinese Han women population evaluated the association of SNPs of ER2 genes, rs1256049 and rs4986938, with CAD. Results of this study revealed that there was no significant association between the genotypes and/or haplotypes of these two SNPs and risk of CAD. Further subdivided age-based analysis in this study revealed significantly lower risk of CAD in AG genotypes of rs4986938 in comparison to homozygotes GG carriers in patients younger than 40 years of age. In contrast, homozygotes AA carriers had higher risk of CAD. They concluded that A allele of rs4986938 SNP would be an important predictor of CAD risk in Chinese women younger than 40 years of age. These correlations were not found in rs1256049 SNP [21]. Another case–control study on Brazilian population with premature CAD revealed a statistically significant association between rs4986938 SNP and CAD and it was introduced as one of the most important independent risk factors for CAD as well as dyslipidemia, elevated levels of triglycerides and apolipoprotein B and low levels of HDL in this research. The homozygote AA genotype was more prevalent in case group in comparison to control group [22]. Similar to these studies, our results showed a significant association between rs4986938 genotypes and CAD; however, with its complexity instead of its occurrence. Therefore, rs4986938 SNP may serve as an independent predictor of higher SS. However, a gender difference was reported regarding this association in different populations.

There are some other researches worked on other SNPs of ER2 gene such as rs1271572. A nested case–control study on Spanish population which was assessed the relationship between three SNPs of ER2 and myocardial infarction (MI) revealed that there was a significant association between rs1271572 SNP and enhanced risk of MI occurrence. This finding was limited to the men, therefore; this study suggested the potential role of gender in genetic variance of ER2 gene polymorphisms in MI patients [23]. Another study performed on American population confirmed a significant association between T allele of rs1271572 SNP and higher risk of MI and CVD, however; this association was just seen in women. Because of these controversial results on the association of ER2 polymorphisms and the risk of CVD occurrence and severity, further studies with larger sample sizes and assessments of patients with different ethnics and genetics would be necessary [20].

The exact mechanism by which ER signaling and ER gene polymorphism play a role on CAD pathogenesis is uncertain. Recent studies revealed that estrogen could induce anti-fibrotic effects in the heart that would be occurred through the ER2 [2]. Results of an in vivo animal study revealed that activation of ER2 could stop the effects of angiotensin II (Ang-II) and endothelin-1 (ET-1) pro-fibrotic signaling thus prevent further cardiac fibrosis. Ang-II and ET-1 could induce cardiac fibrosis through the inversion of fibroblasts to myofibroblasts and also through the induction of transforming growth factor-β1 (TGFβ1) which is a known cardiac fibrosis inducer. Estrogen and ER2 agonists could stop TGFβ1 action via cAMP and protein kinase A pathway [3, 24]. Also previous studies revealed that estrogen administration could enhance ER2 transcript expression which induces its anti-fibrotic effects [25]. It has been reported that ER2 gene overexpression would be a predictive factor for heart function improvement and survival in patients who had a recent history of MI both in men and women [26]. Results of another study on the role of ER1 and ER2 gene expression on the occurrence of neovascularization after ischemic heart disease revealed that both ER1 and ER2 (with the dominance of ER1) might have a pivotal role in estrogen-induced endothelial progenitor cells (EPCs) mobilization and further protection of cardiac function following a recent MI [27]. Another suggestive cardioprotective mechanism of ER2 gene would be through the induction of eNOS expression and further vasodilation in cases of ischemia/reperfusion (I/R) injury, especially in women [28]. These researches emphasizes the fact that in addition to the estrogen plasma level and the extent of ER1 and ER2 gene expression, evaluation of ER1 and ER2 genetic variance and polymorphisms would be helpful as predictive measures for identification of high risk patients for CVD and also their prognosis.

Because of the controversies around the role of ER2 SNPs in CVD in different populations, further larger investigations would be necessary in order to confirm our observed association between rs4986938 polymorphism and SS.

### Study limitations

The possible limitations of our study were small sample size and unequal number of participants according to their gender (men and women). Also, it is suggested to evaluate the plasma estrogen levels of patients in combination with investigation of ER2 gene polymorphisms in future studies.

## Conclusions

Besides to the estrogen level, the genetic variation of its receptors, ER1 and ER2, might play an important role in the pathogensis, the severity and the complexity of CAD. According to our results, rs4986938 polymorphism of ER2 gene may assert a pivotal role in the severity of CAD in particular in men; however, this assumption needs to be proved in higher population studies.

## Supplementary Information

**Additional file 1**. A summary of literature review on Estrogen receptors (ER1 and ER2) polymorphisms evaluation in cardio-cerebrovascular diseases.

**Additional file 2**: The histogram of the SYNTAX score in the study group.

**Additional file 3: Fig. S1.** Un-cropped figure 1 A.

**Additional file 4: Fig. S2.** Un-cropped figure 1B.

## Data Availability

All data generated or analysed during this study are included in this published article.

## Abbreviations

ER1: Estrogen receptor alpha
ER2: Estrogen receptor beta
SS: SYNTAX score
CVD: Cardiovascular disease
CAD: Coronary artery disease
CHD: Coronary heart disease
MVD: Multi-vessel disease
LMD: Left main coronary artery disease
MI: Myocardial infarction
CKD: Chronic kidney disease
DM: Diabetes mellitus
Ang-II: Angiotensin II
ET-1: Endothelin-1
TGFβ1: Transforming growth factor-β1
EPCs: Endothelial progenitor cells
ACE: Angiotensin converting enzyme
GFR: Glomerular filtration rate
MDRD: Modification of diet in renal disease
GPR30: G-protein-coupled estrogen receptor 30
SNP: Single nucleotide polymorphism
IMT: Intima-media thickness
LDL: Low density lipoprotein
PCR: Polymerase chain reaction
