# Supplementary material for: The association between estrogen receptor 2 gene polymorphism and complexity of coronary artery disease: an analysis in elective percutaneous coronary intervention patients
Source: BMC Cardiovasc Disord. 2021 Jun 4;21:275. doi: 10.1186/s12872-021-02088-1 (PMC8176575; doi:10.1186/s12872-021-02088-1)
Supplement: Supplementary file 1 — Additional file 1. A summary of literature review on Estrogen receptors (ER1 and ER2) polymorphisms evaluation in cardio-cerebrovascular diseases. [file 12872_2021_2088_MOESM1_ESM.docx]

**Supplementary Table 1.** A summary of literature review on Estrogen receptors (ER1 and ER2) polymorphisms evaluation in crdio-cerebrovascular diseases.

| **First author** | **Study location** | **date of publication** | **sample size** | **Name of SNPs** | **Study design** | **Findings** | **Ref.** |
| --- | --- | --- | --- | --- | --- | --- | --- |
| MM Wu | Taiwan | 2010 | 760 | rs2228480, rs3798758 | Evaluation of the association between ER1 single nucleotide polymorphism (SNP) and Intima-media thickness (IMT) in carotid artery, an important predictor of CVD, in Taiwanese women population. | Results of this research demonstrated a significant association between rs2228480 and rs3798758 and the IMT of carotid artery in women. According to this article, women with rs2228480 polymorphism had an increased risk for IMT of carotid artery, therefore; were more prone to develop atherosclerotic disorders such as ischemic stroke.  Results from multiple linear regression analyses showed significant associations of SNPs rs2228480 and rs3798758 with the carotid IMT values in women but not in men. Women with SNP rs2228480 A/A genotype had a 7.1% increase in IMT values versus the other genotypes combined. Women with rs3798758 CA+AA combined genotypes, had less carotid IMT measures (about 2.9%) compared to those carried CC genotype | [1] |
| T Kunnas | Finland | 2010 | 2225 | rs2234693 (PvuII),  rs9340799 (XbaI) | Evaluation of the relationship between ER1 gene polymorphism and the risk of coronary heart disease (CHD) and stroke in Finnish population. | Results of this research revealed the significant association of rs2334693 polymorphism with the higher risk of CHD in men. On the other hand, this study failed to confirm any significant association between ER1 gene polymorphism and the occurrence of ischemic stroke.  In men, the minor CC genotype of the rs2234693 polymorphism contributed to a higher risk of CHD, compared to those with the T-allele. Haplotype analysis revealed that men with haplotype CA of variants of rs2234693 and rs9340799 had even higher risk for CHD. | [2] |
| S Demissie | Data extracted from the Framingham Heart Study’s offspring component (FOS) | 2006 | 854 | rs2077647,  rs2234693 (PvuII),  rs9340799 (XbaI) | Evaluation of the association between ER1 gene polymorphism and low density lipoprotein (LDL) metabolism in women. | Results of this study reported that there was a significant association between ER1 gene polymorphism and low density lipoprotein (LDL) metabolism, an important predictor of atherosclerosis and CVD, in women.  The S allele of the ESR1 microsatellite (ESR1 TA) repeat and the T allele of rs2234693 are associated with a more atherogenic lipoprotein pattern including higher levels of small LDL particles as well as smaller LDL and HDL sizes. Similar results were obtained in haplotype analyses, in which the TA[S]–c.30T–c.454-397T–c.454-351A haplotype was associated with significantly higher concentrations of small LDL particles compared to the TA [L]–c.30C–c.454-397C–c.454-351G haplotype. | [3] |
| I Peter | Data extracted from the Framingham  Offspring Study (European descent) | 2009 | 1261 | rs2077647,  rs2234693, rs9340799 | Evaluation of the relationship between ER1 and ER2 gene polymorphisms and increased arterial stiffness as well as enhanced wave reflection. | Results of this study demonstrated a strong relationship between ER1 and ER2 gene polymorphisms and enhanced wave reflection as a cardiovascular disease risk factor.  Among smokers, those who were homozygous for rs2234693 C allele had a 40% higher augmented pressure and 33% greater augmentation index than carriers of one or two major alleles. A significant association between ESR1 rs2234693 polymorphism and lipoprotein fraction concentrations has been found in this cohort. Among smokers, female carriers of ER1 rs2234693 TT genotype had higher levels of small LDL particle and lower sizes of LDL particles than non-carriers, whereas no such association was detected in non-smokers. | [4] |

**References:**

1. Wu MM, Hsieh YC, Lien LM, Chen WH, Bai CH, Chiu HC, et al. Association of estrogen receptor {alpha} genotypes/ haplotypes with carotid intima-media thickness in Taiwanese women. Angiology. 2010;61(3):275-82.

2. Kunnas T, Silander K, Karvanen J, Valkeapaa M, Salomaa V, Nikkari S. ESR1 genetic variants, haplotypes and the risk of coronary heart disease and ischemic stroke in the Finnish population: a prospective follow-up study. Atherosclerosis. 2010;211(1):200-2.

3. Demissie S, Cupples LA, Shearman AM, Gruenthal KM, Peter I, Schmid CH, et al. Estrogen receptor-alpha variants are associated with lipoprotein size distribution and particle levels in women: the Framingham Heart Study. Atherosclerosis. 2006;185(1):210-8.

4. Peter I, Kelley-Hedgepeth A, Huggins GS, Housman DE, Mendelsohn ME, Vita JA, et al. Association between arterial stiffness and variations in oestrogen-related genes. Journal of Human Hypertension. 2009;23(10):636-44.
