# Supplementary figures and images for: The association between estrogen receptor 2 gene polymorphism and complexity of coronary artery disease: an analysis in elective percutaneous coronary intervention patients
Source: BMC Cardiovasc Disord. 2021 Jun 4;21:275. doi: 10.1186/s12872-021-02088-1 (PMC8176575; doi:10.1186/s12872-021-02088-1)

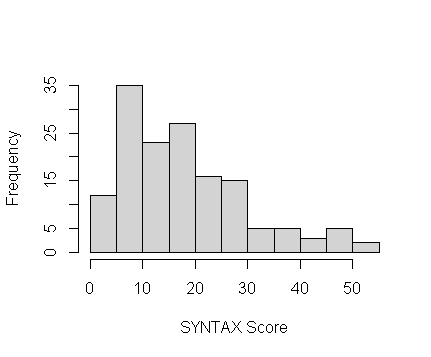

Supplement: Supplementary file 2 — Additional file 2: The histogram of the SYNTAX score in the study group. [file 12872_2021_2088_MOESM2_ESM.tiff]

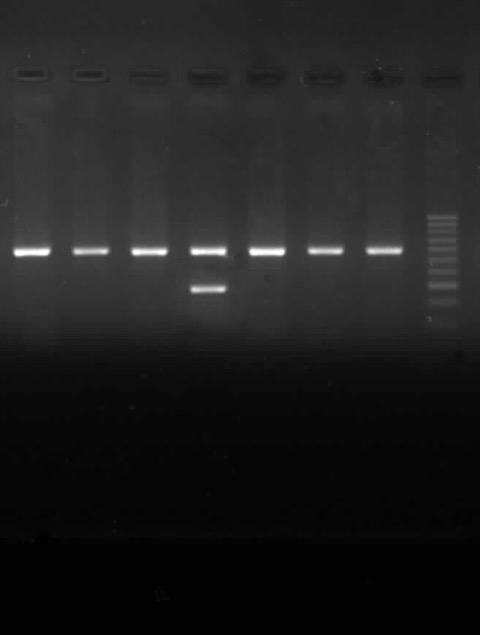

Supplement: Supplementary file 3 — Additional file 3: Fig. S1. Un-cropped figure 1 A. [file 12872_2021_2088_MOESM3_ESM.jpeg]

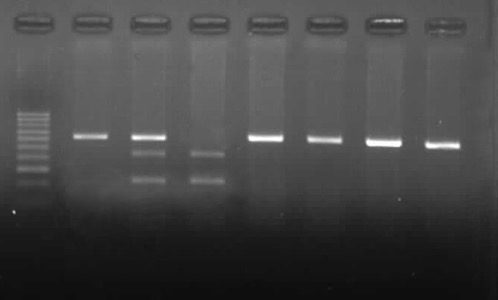

Supplement: Supplementary file 4 — Additional file 4: Fig. S2. Un-cropped figure 1B. [file 12872_2021_2088_MOESM4_ESM.jpeg]
